# Disentangling Migratory Routes and Wintering Grounds of Iberian Near-Threatened European Rollers *Coracias garrulus*

**DOI:** 10.1371/journal.pone.0115615

**Published:** 2014-12-31

**Authors:** Juan Rodríguez-Ruiz, Javier de la Puente, Deseada Parejo, Francisco Valera, Miguel A. Calero-Torralbo, José M. Reyes-González, Zuzana Zajková, Ana Bermejo, Jesús M. Avilés

**Affiliations:** 1 Departamento de Ecología Funcional y Evolutiva, Estación Experimental de Zonas Áridas (CSIC), Almería, Spain; 2 Área de Estudio y Seguimiento de Aves, SEO/BirdLife, Madrid, Spain; 3 Área de Zoología, Departamento de Anatomía, Biología Celular y Zoología, Facultad de Ciencias, Universidad de Extremadura, Badajoz, Spain; 4 Department of Animal Biology/Vertebrates, Faculty of Biology, University of Barcelona, Barcelona, Spain; Università degli Studi di Milano-Bicocca, Italy

## Abstract

Long-distance migrants are suffering drastic declines in the last decades. Causes beneath this problem are complex due to the wide spatial and temporal scale involved. We aim to reveal migratory routes, stopover areas, wintering grounds, and migratory strategies for the most southwestern populations of the near-threatened European Roller *Coracias garrulus* in order to identify conservation key areas for the non-breeding stage of this species. To this end, we used tracking data from seven satellite transmitters fitted to birds breeding in different populations throughout the Iberian Peninsula and four geolocators fitted to individuals in a southeastern Iberian population. Precise satellite data were used to describe daily activity patterns and speed in relation to the main regions crossed during the migration. Individuals from the most southwestern Iberian populations made a detour towards the Atlantic African coast whereas those from northeastern populations followed a straight north-to-south route. We identified important stopover areas in the Sahel belt, mainly in the surroundings of the Lake Chad, and wintering grounds on southwestern Africa farther west than previously reported for the species. Concerning the migratory strategy, satellite data revealed: 1) a mainly nocturnal flying activity, 2) that migration speed depended on the type of crossed habitat, with higher average speed while crossing the desert; and 3) that the migration was slower and lasted longer in autumn than in spring. The studied populations showed weak migratory connectivity, suggesting the confluence of birds from a wide range of breeding grounds in a restricted wintering area. Therefore, we suggest to target on defining precisely key areas for this species and identifying specific threats in them in order to develop an appropriate global conservation programme for the European Roller.

## Introduction

Migrant birds are highly vulnerable, as they must cross many different habitats while performing their year-round life cycle. Consequently, migrants are liable to be affected by multiple ecological and social challenges in diverse places and occurring at different times [1]. Wide continental analyses have revealed that migrant birds are involved in a sustained decline of their breeding populations in Europe, which is even more drastic for long-distance migrants [2], [3]. Therefore, a major challenge for conservation biologists is to investigate whether the observed decline of migrants is related to the loss of breeding or winter habitats, heightened mortality during migration, or some combination of these [4]. Thus, conservation of migratory birds depends largely on the identification of key areas and threats through the migratory pathway and in their wintering grounds as well as the examination of migratory connectivity among populations [5], [6]. This urge, together with the decreasing size and cost of tracking technologies have fuelled a growing interest for studying the non-breeding stages of many medium and small-sized migrant birds [7]–[11], even though the total harmlessness of these techniques is debatable [12]–[15].

In this context, identifying migratory strategies is also essential to understand how birds may optimize energy expenditure in relation to foraging and meteorological conditions they experience during their journey [16]. For instance, nocturnal migration allows diurnal foragers to refuel during daylight for travelling by night, when they might evade predators and worst diurnal atmospheric conditions, such as extremely high temperatures and/or air turbulences [17]. Alternatively, diurnal migration may be beneficial in terms of orientation, allowing birds to find best habitats to stop and forage, or foraging while flying [18], and avoiding the negative consequences of sleep deprivation [19]. Migratory behaviour may also vary between and within species in response to habitat characteristics that pose major challenges at a wide spatial scale. For instance, some trans-Saharan migrants have adapted their pace by increasing their speed and lowering the number of stops [20], [21], and others by choosing an alternative route [22].

The European Roller *Coracias garrulus* (Roller, hereafter) has suffered a global decline of its breeding population of about 30% in the past few decades [23], classing this species as near-threatened. Indeed, this decline is among the most drastic ones for Afro-Palaearctic migrant farmland birds wintering in open savannahs [2]. The knowledge about the migration routes and wintering grounds of Rollers is scant as it comes from a few ringing recoveries of central and eastern European birds at a few African places [24]–[26]. The first serious attempts of revealing the migratory route of western Rollers' populations combined field observations, a low number of ringing recoveries and the occasional report of corpses [27]. Based on this disjointed information up to four different routes have been hypothesized: 1) diagonal crossing of the Sahara Desert to eastern Africa [28], [29]; 2) a detour to the east crossing south towards Tibesti-Uganda [30]; 3) a north-south cross of the Desert to Niger [27] or; 4) a detour to the west passing through Senegal [31], [32]. Recently, deployment of geolocators in southern France and Portugal based on three and two individuals respectively, show two alternative routes to reach wintering grounds in southwestern Africa in Angola: the former following a straight track across the Mediterranean Sea and Sahara Desert [33], and the later taking a more western route [34]. However, these patterns were based on less accurate geolocators, which precluded a precise report of spring migration, and on individuals from a single breeding locality. In this work, by deploying satellite transmitters and geolocators on adult breeding individuals from different Iberian populations, we describe in detail for the first time migratory routes and wintering grounds of the most south-western European populations of this near-threatened species. Moreover, we describe its migratory strategy along different stages of the journey. In addition, we explored migratory connectivity for Iberian Rollers by studying the spatial distribution of individuals in their breeding and wintering grounds.

## Materials and Methods

Adult Rollers were trapped using nets during the late incubation stage or when they had small nestlings in 2012 and 2013 breeding seasons (June-July) in Spain. Birds were ringed, measured and weighted and most of them were also sexed by molecular methods [35] (Table 1). 5-g solar-powered PTT-100 satellite transmitters (Microwave Telemetry Inc., Columbia, MD, USA) were deployed on twelve individuals as a backpack using a ribbon Teflon harness [36], although we only recorded seven birds initiating their migration due to death or device malfunction of the other five birds: one female in the southeast (Granada, hereafter PTT-GR), two males and one unsexed individual in the middle zone (Madrid, PTT-M; Ciudad Real, PTT-C; and Badajoz, PTT-B) and three males in the northeast (Lleida, PTT-L; Girona, PTT-Ģ and Huesca, PTT-H) (Table 1).

**Table 1:** Migration timing and distances of eleven tracked European Rollers.

	Breeding Location	Sex	Departure Date	Arrival to Lake Chad Basin	Departure from L. Chad Basin	Arrival Date	Wintering Location	Duration (days)	Travelling Days	Total Distance (km)	Avg Speed (km/d)	Stopovers (locations)
*Autumn Migration*												
PTT-M	Madrid, 40°16′ N, 4° 06′ W	Male	04 Aug 2012	18 Sep 2012	23 Oct 2012	01 Dec 2012	Namibia	119	37.5	9,547	254	11
PTT-L	Lleida, 41°49′ N, 0°37′ E	Male	06 Sep 2012	23 Sep 2012	03 Nov 2012	21 Nov 2012	Botswana	76	20.5	8,338	406	7
PTT-C	Ciudad Real, 39°19′ N, 3°18′ W	Male	25 Aug 2012	23 Sep 2012	24 Oct 2012	03 Dec 2012	Botswana	100	36.5	10,095	276	12
PTT-GR	Granada, 37°19′ N, 3°02′ W	Female	04 Aug 2012	07 Oct 2012	26 Oct 2012	17 Nov 2012	Namibia	105	36.5	9,986	273	9
PTT-G	Girona, 42°17′ N, 3°07′ E	Male	12 Sep 2013	Not reached	-	Not reached	Not reached	-	8.5*	2,763*	329	1*
PTT-H	Huesca, 41°47′ N, 0°11′ E	Male	8 Sep 2013	19 Oct 2013	19 Nov 2013	22 Dec 2013	Namibia	105	28	8,996	318	12
PTT-B	Badajoz: 38°42′ N, 6°54′ W	Unidentified	27 Aug 2013	5 Oct 2013	21 Oct 2013	13 Nov 2013	Angola	78	40	8,651	218	5
GLS-1	Almería, 37° 05′N, 2° 21′W	Female	21 Jul 2012	27 Sep 2012	16 Oct 2012	31 Oct 2012	Angola	102	22	9,380	426	4
GLS-2	Almería, 37° 05′N, 2° 21′W	Female	19 Aug 2012	18 Oct 2012	29 Oct 2012	09 Nov 2012	Namibia	82	21.5	9,471	348	4
GLS-3	Almería, 37° 05′N, 2° 21′W	Male	21 Jul 2012	16 Sep 2012	26 Oct 2012	07 Nov 2012	Botswana	109	24	9,406	392	5
GLS-4	Almería, 37° 05′N, 2° 21′W	Female	19 Aug 2012	27 Sep 2012	01 Nov 2012	19 Nov 2012	Namibia	92	37.5	8,719	232	2
								*101 (20.5)*	*32.25 (14.75)*	*9,393 (739.75)*	*348 (141.5)*	*6 (6.25)*
*Spring Migration*												
PTT-H	Huesca, 41°47′ N, 0°11′ E	Male	13 Mar 2014			-	Namibia	-	-	-	-	-
PTT-L	Lleida, 41°49′ N, 0°37′ E	Male	10 Mar 2013			12 May 2013	Botswana	63	23	9,884	426	7
GLS-2	Almería, 37° 05′N, 2° 21′W	Female	23 Feb 2013			06 May 2013	Namibia	72	19.5	10,679	545	3
GLS-3	Almería, 37° 05′N, 2° 21′W	Male	10 Feb 2013			14 Apr 2013	Botswana	63	18	8,712	484	2
GLS-4	Almería, 37° 05′N, 2° 21′W	Female	01 Apr 2013**			06 May 2013	Namibia	35	20.5	7,999	390	1
								*63 (9.25)*	*20 (2)*	*9,298 (1,549)*	*455 (82.25)*	*2.5 (2.25)*

Total distance refers to the entire migratory journey estimated as the sum of migration segments length (one point per day). Duration is the time (in days) spent by each individual from their departure from breeding sites to their arrival to wintering grounds (or vice versa for spring migration), including time spent in stopovers (which are excluded in Travelling Days). Average flight speed is the total distance divided by the number of travelling days excluding days spent in stopovers. Last rows values are the median and interquartile range (in parentheses) per season (*Route incomplete. Excluded from median and IQR) (**Equinox interference does not allow to determine precise departure date. Thus date shown is considered the latest possible departure date).

Satellite transmitters were programmed with an about 8-h ON/ 15-h OFF duty cycle. Cycles started at different time of the day allowing to get a rough representation of the complete daily pattern of activity. For each duty cycle, the satellite transmitter sent a variable number of locations of different quality. High quality locations (classified according to their accuracy as LC 3 (up to 250 m), 2 (up to 500 m), 1 (up to 1.5 km); [37]) were preferentially used. However we relied on lower quality locations (>1.5 km) to define important data points (i.e. precise arrival or departure dates) when higher quality data were not available. We got (mean ±SD) 1127±563 locations per device on average, of which a 46.2±17.4% were high quality data (LC 3, 2 and 1). To describe individual migratory routes we built a dataset by selecting one of the highest quality locations per hour and individual. PTT-L reached its breeding area in 2013 and, therefore, provided us with a complete record of its annual cycle. PTT-H performed its autumn migration and was still recording by the time this work was done. The signal of PTT-G was lost at some point during its autumn migration, whereas PTT-M and PTT-C signals were lost during their spring migrations. Finally, the signals of PTT-GR and PTT-B were lost once they arrived at their wintering quarters. Moreover, during the 2012 breeding season, 12 adults settled in a population located in the southeast of Spain (Almería) were tagged with 2.5 gram light-level geolocators (model MK4490C manufactured by Biotrack Ltd., United Kingdom). Geolocators use ambient light to estimate latitude and longitude (2 positions per day), determined by day and night lengths and time of local midday or midnight, respectively [38]. Although precision of locations is considerably lower for geolocators than satellite transmitters, they have proved to be an efficient technique for assessing long-distance movements for small and medium-sized birds [9], [10], [39], [40], including Rollers [33], [34]. Half of the geolocators were fitted with a ribbon Teflon leg-loop harness [41] and half with a backpack harness [36]. Four birds equipped with geolocators were recaptured the next year, two of each attachment design (named as GLS-1 to GLS-4) (Table 1). For unknown reason GLS-1 stopped recording locations at the wintering grounds.

Geolocators were calibrated for one week before deployment at a location close to breeding sites. Light-level curves were generated and supervised using BASTrack software [42], inferring latitude from length of daylight and darkness periods, and longitude from local time for midday and midnight. We used a threshold value of 2, and values of solar angle obtained for each device from calibration period (angles ranging from −4.9 to −4.5). A confidence value was assigned to each dusk/dawn according to interferences and uncertainty in the light curve and timing.

After obtaining geographic coordinates data, we evaluated the reliability of each position according to days from equinox and confidence values. An iterative forward/backward averaging velocity filter was applied to remaining positions [43]. The speed limit index was set as the 95^th^ percentile of the speed index [44]. After filtering, we discarded on average 34% of positions for each individual.

Location data from geolocators collected between the 1^st^ September and 12^th^ October and between 28^th^ February and 10^th^ April were estimated only from longitude, as estimations of latitude during the equinoxes are highly imprecise. In addition, latitudinal deviations are higher when locations are close to the equator [45]. Therefore, when departure or arrival from one stopover or wintering area was influenced by equinox, producing unreliable locations, we trusted on previous or late reliable positions to estimate latitude. Next, we looked on their corresponding longitude coordinate to determine movement according to position variation or stabilization in the horizontal axis. If the period of longitude stabilization extended beyond the equinox, we assumed that no movement happened either in longitudinal and latitudinal axis during that period. Hence, departure or arrival date was assigned when longitude varied. However, results derived from these periods and latitudinal range should be considered as approximations.

Onset of migration was identified by abrupt changes in movement pattern, directionality and stability of travelled distance following Limiñana *et al.* [46]. End of migration was identified when we detected a stop longer than 10 days once the bird had crossed the equator. From satellite data, stopovers were assigned when we detected less than 20 km of displacement in at least 24 hours. From geolocators data, stopovers were identified when at least five consecutive points were included within an area with a diameter of 300 km (given that the average error ±SD of geolocators is 185±115 km [42]). For clarity in the maps, we used the average values of latitude and longitude coordinates from locations assigned to each stopover. To represent the wintering grounds of satellite-tracked birds, we calculated average coordinates per individual using only one random of the highest quality positions for each 24 hour period. For geolocators, we used Home Range Tools for ArcGIS 9.3 [47] and estimated kernel density areas which comprised 50, 70 and 90 percent of the wintering locations.

### Migratory strategy

From the data set extracted from satellite transmitters we selected pairs of high quality locations for each individual (excluding inactive locations corresponding to stopovers and wintering grounds) yielding a subset of 132 segments of 1 to 8 hours duration. As distance travelled and time lapse for each segment are known, we were able to estimate the speed of flight of each bird in these segments. This allowed us to study flight activity of each bird in relation to time of day and region overflown. We classified every segment attending to a threshold of 5 km/h [48] into *stationary*, when speed was below 5 km/h, and *travelling*, when speed was above 5 km/h. We also classified segments according to latitude in four regions: Sahara Desert (from Mediterranean Sea to 19°N), Sahel (from 19°N to 7°N), Equator (from 7°N to 15°S) and Southern Savannah (15°S to wintering grounds). Finally, segments were classified according to light conditions as daylight and darkness in relation to dusk and dawn local hours.

### Speed of migration

**Figure 1:**
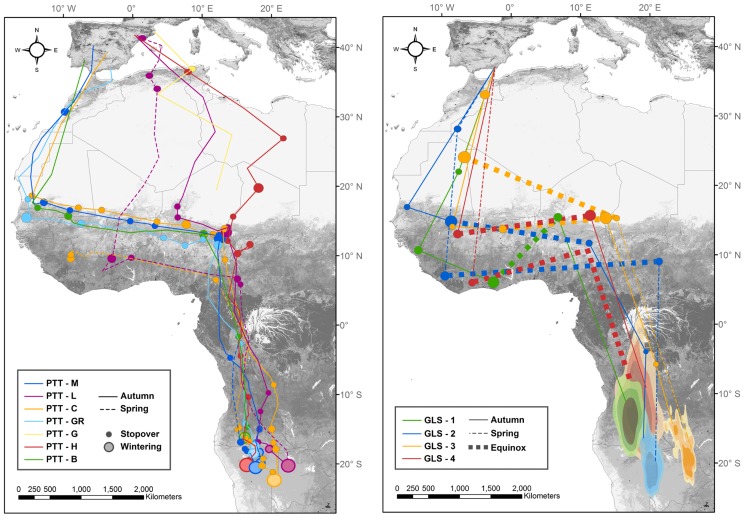
Migratory routes, stopover sites and wintering grounds of southwestern European Rollers. Tracks recorded by satellite tranmitters (left) and geolocators (right) between breeding sites in the Iberian Peninsula and the wintering grounds (blue dots and blue areas, respectively) in southwestern Africa. Red and green colours correspond, respectively, to autumn and spring migrations. Dots represent stopovers, and their sizes are relative to time spent (the longer the period, the bigger the spot). Dotted lines indicate uncertainty provoked by the equinoxes. Locations assigned before and after dotted lines are partially based on longitudinal data and should be considered as approximations. Geolocators data for latitudes close to the equator have high associated deviations and thus should be interpreted carefully [45]. Wintering grounds for satellite-tracked birds were located by calculating average coordinates, whilst for geolocators they were calculated by kernel density estimation for 50, 70 and 90% of the locations.

To estimate the average speed during migration we calculated duration and distance for each migratory step for both geolocator and satellite-tracked birds. One step was defined as the movement between two consecutive stops. Duration was calculated using the starting and ending locations of the movement. Distance was differently estimated depending on the type of stop: for breeding grounds we used the known capture location; for stopovers we used the average location calculated from high quality data; for wintering grounds we used the first high quality location as the final destination in autumn migration and the last one as the starting point in spring migration in order to avoid likely itinerancy during winter [49]. Speed of migration from geolocators data around the equinoxes was derived using the average of those locations within the stopover that were not affected by equinox (thus we are assuming that latitudes does not vary while longitude is constant), and duration was estimated from changes in longitude. Each step was classified in regions as indicated for short segments. As steps can be long and include more than one region (Fig. 1), we assigned the step to the region that represented a major proportion of its length. Movements outside Africa (i.e. across the Mediterranean Sea or the Iberian Peninsula) were discarded in analyses of differences among regions.

### Statistical methods

Statistical analyses were performed with the software SAS, version 9.2 and R 2.15.3 [50]. Mann-Whitney tests were used to analyze differences in duration and speed of migration between autumn and spring seasons. We fitted a generalized linear model by stepwise procedure to model variation in Roller flight activity based on segments as a binomial dependent variable (stationary *vs*. travelling, logit link function; PROC GLIMMIX procedure in SAS) in relation to time of day (daylight *vs.* darkness) and overflown region as fixed terms. The interaction between these two factors was also entered to test whether patterns of flight activity between day and night changed over regions. A previous analysis in which we entered individual identity as a random intercept to this model to control for non-independence of segments from different individuals revealed that the variance component for individual identity was zero and, thus, that individual identity could/should be removed from the model [51]. Results remained the same after the removal of this random intercept. The square-root transformation of speed data for migratory steps was fitted to a general linear mixed model to explore its variation in relation to region using bird identity as a random factor. Analyses were done separately for data based on satellite transmitters and geolocators due to their different error and accuracy. Post-hoc Scheffé multiple comparison tests were performed to look for differences in speed between regions.

To study migratory connectivity we have followed a method similar to the one proposed by Ambrosini et al. [52]. A Mantel test was performed to explore the correlation between distance matrices of breeding and wintering areas of tracked birds using the R package ‘ecodist’ [53]. Distances were measured along loxodromes and were calculated using centroids to represent wintering areas, and locations of capture for breeding grounds. High level of correlation between distance matrices between breeding and wintering locations would suggest high migratory connectivity. The significance of Mantel test was checked using permutations with estimations of p-values after 10000 randomizations. Although ringing recoveries may provide very useful information on this respect, we confirmed that there are not reported ringing recoveries of Spanish rollers outside Spain by checking the Spanish Ringing Scheme database (SEO/BirdLife, http://www.anillamientoseo.org/).

### Ethic Statement

The deployment of geolocators and satellite transmitters did not take more than 20 minutes and had no obvious effects on the reproduction of tagged birds. This study was approved by the relevant authorities (regional governments of Andalucía, Aragón, Castilla-La Mancha, Cataluña y Extremadura; research permits CGL2008-00718 and CGL2008-00562). Hence, all necessary permits were obtained for the study, which complied with the national legislation of Spain concerning animal handling and tagging. Study areas are privately owned and permission to use the areas was acquired from the land owners.

## Results

### Migratory routes and wintering grounds

Departure from the breeding areas occurred between the 21^st^ July and the 6^th^ September in 2012 and between 27^th^ August and 12^th^ September in 2013. These interannual differences may be associated to differences among sampled populations, which differ between years (see Table 1), and/or to isolated cases of breeding failure (e.g. the bird that started migration on July 21^st^ failed to raise any fledgling). Crossing to Africa occurred in three ways (Fig. 1). Birds breeding in central Spain headed south towards the Gibraltar Strait. Meanwhile, birds breeding in the south-east of Spain arrived to Africa crossing the Mediterranean Sea usually following a straight line, through the Alboran Sea. The three birds from the northeastern populations, however, crossed the Mediterranean Sea through the Balearic Islands or near them to enter Africa by Algeria and Tunisia, with short stopovers near the coast (3 and 5 days). After reaching Africa, birds either continued southeast through the Sahara Desert or southwest following the coastal line to their first stops in the Sahel Belt in Senegal, Guinea and the southern regions of Mauritania and Mali, where they stayed between 4.8 and 39 days. In two cases, Rollers had first stopped along the southern face of the Atlas Mountain Range in Morocco (7.5 and 19.5 days). We also detected one important stopover in Burkina Faso of 16.5 days. Next, they followed the northern border of the Sahel Belt to the East to reach Chad, Niger and Nigeria, making short stopovers on the way, where they generally spent less than a week, except for the bird GLS-1 that apparently took a southern route to Ghana, where it spent 35.5 days. Bird PTT-L reached Chad from Algeria flying through Tunisia and Libya without any stopover on the way. Bird PTT-H travelled first to a short stopover in the east of Libya (1 day) and turned south to Chad making an important stopover in the north of Chad (26 days). In Chad, Niger and Nigeria, we identified long stopovers for every bird taking place from 16^th^ September to 7^th^ November and staying between 11 and 41 days. Central Iberian birds took 29 and 44 days to complete this first stage of the migration, whereas southeastern birds ranged from 39 to 68 days. On the other hand, northeastern birds spent 17 and 41 days to perform their route. From the long stopover in Sahelian areas in Chad, Niger and Nigeria, every bird headed south-southeast to Angola, Namibia and Botswana between 16^th^ October and 19^th^ November, where they established their wintering grounds (Table 1, Fig. 1). Birds arrived there between 31^st^ October and 22^nd^ December and their stay lasted between 66 and 133 days. We detected previous short stopovers in Angola and Congo in satellite-tracked birds, but in the case of geolocator-tracked birds we were not able to identify any stopover close to the wintering grounds due to their lower precision. Spring migration information corresponds only to year 2013 for birds tagged in 2012, as the last date available for those tagged in 2013 was 16^th^ March 2014. Spring migration started from 10^th^ February to 1^st^ April, when birds headed north-northwest to Central African Republic, Chad and Cameroon to make a short stopover of 3 to 18.5 days (Fig. 1). Next, they headed to the West mainly following the southern border of the Sahel Belt to stop in Ivory Coast, Liberia, Ghana and Guinea for 13.5 to 24.5 days. From these points they changed their directions to the north, heading towards the Alboran Sea to arrive to the Iberian Peninsula in cases of birds GLS-2 and GLS-4 and towards north Algeria in the case of PTT-L, to cross again the Mediterranean Sea to Menorca Island, where it turned west to its breeding ground. Bird GLS-3 travelled by a northern route from Chad to Mauritania, where it stayed 37.5 days, and then joined the western route to the Alboran Sea. No bird entered the Iberian Peninsula through the Gibraltar Strait, but we lacked returning information from Rollers from the most western breeding populations. Rollers arrived at their breeding grounds between the 14^th^ April and 12^th^ May.

Comparison of duration and speed of migration between autumn and spring seasons revealed that autumn migration lasted significantly longer when we considered time spent in stopovers (Table 1, Mann-Whitney test: *Z*-adjusted  = 2.83, *P* = 0.005) and after excluding it (Mann-Whitney test: *Z*-adjusted  = 2.33, *P* = 0.020). Consequently, autumn migration was significantly slower than spring migration (Table 1, Mann-Whitney test: *Z*-adjusted  = −2.42, *P* = 0.016), although the number of stopovers did not significantly differ between the two migratory periods (Mann-Whitney test: *Z*-adjusted  = 1.84, *P* = 0.07).

### Migratory strategy: Flight activity in relation to daily time and region

**Figure 2:**
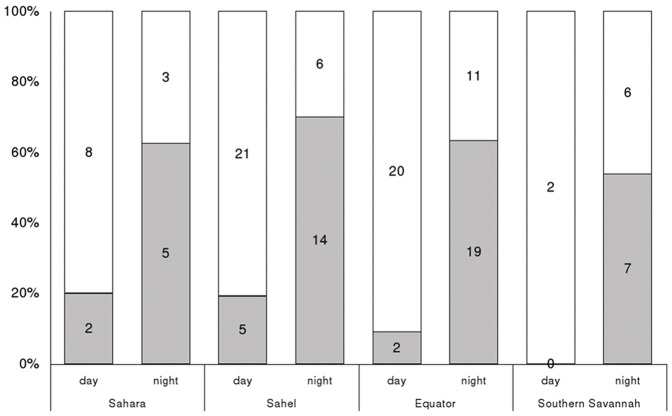
Travelling activity during migration of Rollers. Percentage of segments flying faster than 5 km/h in relation to time of day and overflown region. Numbers on bars indicate the number of segments in each category (grey for travelling segments, white for stationary segments).

Analysis of segments revealed that travelling movements (>5 km/h) were significantly more frequent during darkness than during daylight (time of day effect: *F*_1,126_ = 27.80, *P*<0.001, Fig. 2). This pattern did not vary between the four regions (time of day* region effect*: F*_3,123_ = 0.18, *P* = 0.91; region effect: *F*_3,126_ = 0.59, *P* = 0.62; Fig. 2). Estimated speed in segments was (median [range]) 9.87 [0.12 to 84.31] km/h during the night and 1.31 [0.09 to 46.42] km/h during daylight hours.

### Overall speed of migration

**Figure 3:**
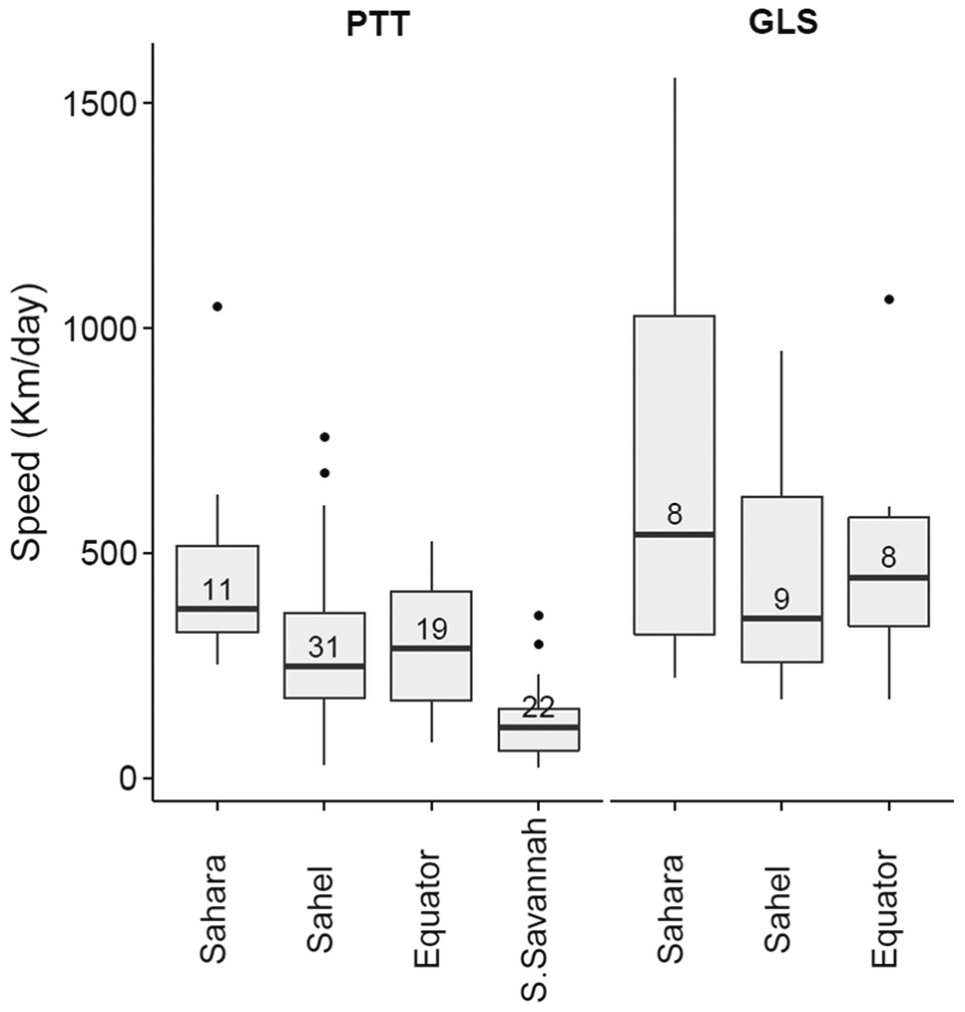
Migration speed of Rollers in relation to region. Box plot showing median levels of migration speed (km/day) in relation to crossed region, separated by tracking method: satellite transmitters (PTT) and geolocators (GLS). Boxes show interquartile ranges (IQR). Bars represent maximum and minimum values within 1.5 IQR. Dots represent outliers (outside 1.5 IQR). Numbers inside boxes represent sample size (number of steps between consecutive stops, given non-significance of the random factor *bird identity*; see Results).

Using the data received from satellites, Rollers travelled at a different speed depending on the overflown region (region effect: *F*_3,73_ = 15.06, *P*<0.001; bird identity effect: *Z* = 0.41, *P* = 0.34, Fig. 3). In particular, we found differences between Sahara and Sahel (Scheffé test: *P* = 0.013) and between Southern Savannah compared to other regions (Scheffé tests S. Savannah – Sahara: *P*<0.001; S. Savannah – Sahel: *P*<0.001; S. Savannah – Equator: *P*<0.001), but they were not significant when comparing Sahel and Sahara with Equator (Scheffé tests: *P* = 0.07 and *P* = 0.96, respectively). Analyses based on less precise geolocators revealed a similar but not significant trend (region effect: *F*_2,19_ = 1.39, *P* = 0.27; bird identity effect: *Z* = 0.76, *P* = 0.22).

### Migratory Connectivity

Ten birds were used to build the distance matrices as one individual did not reach the wintering area. Differences in distance among breeding locations did not significantly relate with differences in distance among wintering locations (r = −0.22, *P* = 0.91).

## Discussion

### Migratory routes and wintering grounds

Our results show that Iberian Rollers migrate to Africa using two alternative strategies (Fig. 1). This seems to indicate the existence of a western limit, or a cline, which separates these different strategies. At an intermediate level of this cline would be the southeastern population of Almería which make a moderate western detour but then cross the Sahara desert directly. However, all birds from this population were tracked with geolocators, and thus we cannot discard the possibility that these results are a consequence of the lower precision of this tracking technique compared to satellite-tracking. More detailed sampling is needed to confirm this split of strategies.

Both western and straight paths converge in the Lake Chad surroundings and, from that point, the last phase of migration is common to every bird, in a continuous flight over the tropical forest to their final wintering destination in the savannahs of southwestern Africa, in Angola, Namibia and Botswana (Fig. 1). Our results also show that Iberian Rollers do not spend the non-breeding period in the savannah belt north of the Sahel, as had been previously suggested [49], [54], and support the hypothesis of a concentrated passage across the equator for land birds that winter at Southern Hemisphere latitudes in Africa [55].

In term of distances, birds using the western route that follows the Atlantic African coast covered 9407 km in average, while the northeastern bird that used the straight route covered 8667 km. The western route leads to a detour twofold longer than the straighter route (41 per cent more distance than the loxodrome versus 23 per cent, respectively). Detours are widespread when facing with strong barriers to minimize risks related to harsh conditions [22]. In this case, it implies a reduction of the travel segment through the desert of 500 km approximately from about 2000 km of which birds using the inland way have to deal with. This route may bring benefits due to lower temperatures and lower risks of dehydration by the proximity of the Atlantic Ocean, even though they have to travel an additional distance to reach their wintering grounds.

Regarding the spring migration, we found different routes among individuals compared to autumn migration. Although birds appear to use the same corridor that they used in the autumn migration to cross the rainforest, the spring route seems to diverge into two alternatives as soon as individuals reach the savannah belt (Fig. 1). Nevertheless, it should be taken into account that details of the spring migratory route are based on 3 geolocators and only 1 satellite transmitter and, therefore, it lacks of the same precision of the description of autumn migration.

### Migratory strategy and behaviour

Our results also show for the first time in this species flight activity during the migratory stage, revealing a different pattern of migration from what was previously thought. Cramp [56] describes the species as a diurnal migrant, but here we demonstrate that migration occurs mainly during the night. This is not surprising considering its diurnal foraging habits (hunting from perches [57]), as well as its flapping flight (thus, they do not depend on thermal air [58]).

Birds covering long distances in each step should spend more time in refuelling to be prepared for the journey and, consequently, they probably would start their migration later than birds which use a route that allows them to stop for rest and forage frequently [59]. This hypothesis is supported by our data, showing a later onset for northeastern Iberian Rollers (all departures were in September, Table 1), whereas southern Rollers started their migration between July and August (Table 1). Nevertheless, this delay does not happen in the French populations, where the onset of migration occurred in mid to end of July [33], which could be due to differences in environmental conditions in the breeding habitats. Moreover, the time spent in completing the first stage from the breeding grounds to cross through the hardest part of the travel reveals different strategies on crossing ecological barriers. Birds from northeastern populations took relatively shorter time (17 and 41 days) than the rest (median (IQR)) 51 (22) days to reach the Lake Chad Basin. Furthermore, the northern bird that took 41 days to reach the lake Chad Basin only spent 15 days to cross Sahara desert, although it rested 500 km north of Lake Chad before reaching it at day 41 from departure. This contrast may not be due to the simple variation in distances, but also to climatic factors and the physical incapability to rest along the travel stretches over the sea and the desert.

### Migratory Connectivity

A major conservation challenge for studies on migration is to measure migratory connectivity of targeted species [5], [6]. According to the results of the Mantel test the spatial distribution observed in the breeding grounds does not correlate significantly with the distribution of individuals observed in the wintering areas, which would indicate a weak migratory connectivity for Iberian Rollers. In addition, Portuguese and French southern populations use the same wintering area, suggesting that this weak connectivity could occur in a larger scale. This scenario of weak connectivity of roller populations could be an issue of concern because the confluence of individuals from a wide breeding range in a restricted wintering location might lead to a high vulnerability of the species due to any possible threat in this area.

### Implications for conservation

The description of the migratory routes and wintering grounds of the near-threatened Roller highlights the challenge of conserving long-distance Afro-Palaearctic migrants, as many different sites and countries are involved (Fig. 1) [60]. An appropriate conservation strategy may involve the definition of ‘key sites’ as sites where different migratory routes converge and Rollers concentrate together. For southwestern Rollers we could define three key sites, namely the Sahel belt, the Lake Chad and the wintering sites among Angola, Namibia and Botswana to which conservation actions should target. The Sahel seems to be a key factor in the decline of long-distance migrants. This big belt gives shelter to many bird species during the migratory and winter periods, and it has suffered an enormous human population growth in the last half of the twentieth century [61], which has led to the deforestation of several areas and drainage of wetlands in favour of agricultural areas. On the other hand, Lake Chad basin is a crucial stopover for every tracked Roller. This area is also important for other trans-Saharan species that are sustained by grasshoppers and locust pulses [62]–[64], highlighting the concern about the fast rate of degradation of this relevant stopover [65]. Finally, although no major conservation problems seem to be in the wintering grounds, a common problem affecting every key site is the use of pesticides to control locust plagues [61], [66], which would negatively affect Rollers attracted to these agricultural areas following the outbreaks of locusts. Furthermore, other problems such as removal of roost trees [66] or illegal shooting [67] should not be underestimated.

In this scenario, the development of an appropriate global conservation programme for the European Roller would need to focus on the following issues: 1) Defining precisely core populations and the geographical division, if it does exist, for the different migratory routes, and the connectivity between and within them by more exhaustive sampling; and 2) Identifying specific threats at the identified key stopover sites during migration and wintering areas to establish conservation priorities and target objectives in a local scale. Roller shares these problems with many other species [1], [2] but being such a spectacular bird it could act perhaps as a ‘flagship’ species in the aim of detecting conservation concerns for trans-Saharan migrants.
